# Phytochemical Profile, Antioxidant Potential, Antimicrobial Activity, and Cytotoxicity of Dry Extract from *Rosa damascena* Mill

**DOI:** 10.3390/molecules28227666

**Published:** 2023-11-19

**Authors:** Antoaneta Trendafilova, Plamena Staleva, Zhanina Petkova, Viktoria Ivanova, Yana Evstatieva, Dilyana Nikolova, Iliyana Rasheva, Nikola Atanasov, Tanya Topouzova-Hristova, Ralitsa Veleva, Veselina Moskova-Doumanova, Vladimir Dimitrov, Svetlana Simova

**Affiliations:** 1 Laboratory Chemistry of Natural Products, Institute of Organic Chemistry with Centre of Phytochemistry, Bulgarian Academy of Sciences, 1113 Sofia, Bulgaria; viktoria.genova@orgchm.bas.bg; 2 Laboratory Organic Chemistry and Spectroscopy, Institute of Organic Chemistry with Centre of Phytochemistry, Bulgarian Academy of Sciences, 1113 Sofia, Bulgaria; plamena.staleva@orgchm.bas.bg (P.S.); zhanina.petkova@orgchm.bas.bg (Z.P.); vladimir.dimitrov@orgchm.bas.bg (V.D.); 3 Research and Development and Innovation Consortium, Sofia Tech Park, 1784 Sofia, Bulgaria; 4 Faculty of Biology, Sofia University “St. Kliment Ohridski”, 1164 Sofia, Bulgaria; y.evstatieva@biofac.uni-sofia.bg (Y.E.); d.nikolova@biofac.uni-sofia.bg (D.N.); i_rasheva@biofac.uni-sofia.bg (I.R.); nikolana@uni-sofia.bg (N.A.); topouzova@biofac.uni-sofia.bg (T.T.-H.); ralitsa_veleva@biofac.uni-sofia.bg (R.V.); moskova@biofac.uni-sofia.bg (V.M.-D.); 5 Institute of Biophysics and Biomedical Engineering, Bulgarian Academy of Sciences, 1113 Sofia, Bulgaria; 6 Bulgarian NMR Centre, Institute of Organic Chemistry with Centre of Phytochemistry, Bulgarian Academy of Sciences, 1113 Sofia, Bulgaria

**Keywords:** *Rosa damascena* Mill, dry rose extract, phenolics, antioxidant activity, antimicrobial activity, cytotoxicity, cell cycle

## Abstract

Dry rose extract (DRE) obtained industrially by aqueous ethanol extraction from *R. damascena* flowers and its phenolic-enriched fraction, obtained by re-extraction with ethyl acetate (EAE) were the subject of this study. ^1^H NMR of DRE allowed the identification and quantitation of fructose and glucose, while the combined use of HPLC-DAD-ESIMS and HPLC-HRMS showed the presence of 14 kaempferol glycosides, 12 quercetin glycosides, 4 phenolic acids and their esters, 4 galloyl glycosides, 7 ellagitannins, and quinic acid. In addition, the structures of 13 of the flavonoid glycosides were further confirmed by NMR. EAE was found to be richer in TPC and TFC and showed better antioxidant activity (DPPH, ABTS, and FRAP) compared to DRE. Both extracts displayed significant activity against *Propionibacterium acnes*, *Staphylococcus aureus*, and *S. epidermidis*, but showed no activity against *Candida albicans.* Toxicity tests on normal human skin fibroblasts revealed low toxicity for both extracts with stronger effects observed at 24 hours of treatment that were compensated for over the following two days. Human hepatocarcinoma (HepG2) cells exhibited an opposite response after treatment with a concentration above 350 µg/mL for EAE and 500 µg/mL for DRE, showing increased toxicity after the third day of treatment. Lower concentrations were non-toxic and did not significantly affect the cell cycle parameters of either of the cell lines.

## 1. Introduction

Medicinal and aromatic plants (MAPs) are the richest bioresource of phenolic compounds and a promising source of natural antioxidants and antimicrobial agents. Therefore, a comprehensive analysis of the phenolic composition of MAP extracts, together with the evaluation of their antioxidative and antibacterial potential, is essential for the discovery of new valuable products with applications in the cosmetic, pharmaceutical, and food industries.

*Rosa damascena* Mill (Rosaceae family), commonly known as Damask rose, holds a significant and symbolic place in Bulgaria with long-standing uses in food preparation and traditional medicine. Decoction and homemade jam or jelly prepared from rose petals have been used as a diuretic and mild laxative for constipation [1]. Rose water is traditionally used to flavor various desserts such as Turkish delight, rice pudding, and yogurt. In addition to its culinary uses, it has also been recognized as an antiseptic agent, facilitating eye rinsing and oral disinfection. It has been used to relieve conditions such as toothaches and headaches, and promote wound healing and overall skin health [1]. In addition, rose flowers, along with their essential oil, have been recommended for their potential blood-purifying effects and their usefulness in dealing with various health problems. These include relief of menstrual problems, management of depression and nervous stress, and alleviating persistent coughs and bile duct inflammation, among others [1].

Nowadays, *R. damascena* is primarily cultivated in Bulgaria to produce the renowned rose oil, rose water, and rose concrete and absolute. These products have found a wide application in perfumery, medicine, and the food industry [2]. Numerous studies have demonstrated the biological effects of rose products. Thus, decoctions, essential oil, and absolute, methanol, and ethanol extracts of rose petals have exhibited antioxidant activity in different systems [3,4] as well as antimicrobial activity against *S. aureus*, *S. typhimurium*, *B. cereus*, *C. albicans*, *P. aeruginosa*, *P. fluorescens*, etc. [5,6,7,8,9,10,11,12]. Additionally, clinical studies have affirmed the efficacy of herbal mouthwash containing aqueous rose extract in the treatment of recurrent aphthous stomatitis [13]. Various extracts of this plant have been reported in the literature to possess antispasmodic, cardiovascular preventive, anti-inflammatory, antidepressant, diuretic, anti-HIV, and skin protective effects [3,4]. All the activities mentioned above have been attributed to the presence of bioactive components, mainly terpenes, flavonoids, anthocyanins, and phenolic acids [5,12,14,15,16,17,18,19,20,21]. Nevertheless, the composition of the extracts, and consequently their biological effects, can be influenced by several factors, including the origin of the plant material and environmental stresses within the cultivation regions, the process of petal collection, and the extraction methodology [22].

In the scope of this study, our primary objectives were to conduct a comprehensive phytochemical characterization of the dry rose extract that is industrially produced by aqueous ethanol extraction of fresh *Rosa damascena* Mill flowers. Furthermore, we aimed to assess the extract’s antioxidant and antimicrobial properties, in addition to evaluating its cytotoxicity. Additionally, we sought to enhance our understanding by isolating and examining the phenolic-enriched fraction derived from the same extract by subsequent re-extraction with ethyl acetate. Through these investigations, we aimed to gain valuable insights into the chemical composition, potential health-related benefits, and safety profile of this rose extract and its enriched fraction, thereby contributing to a broader understanding of their applications in various domains, including pharmaceuticals, cosmetics, and functional foods.

## 2. Results and Discussion

### 2.1. Phytochemical Characterization

#### 2.1.1. Qualitative and Quantitative Determination of Glucose and Fructose by ^1^H NMR Spectroscopy

**Figure 1:**
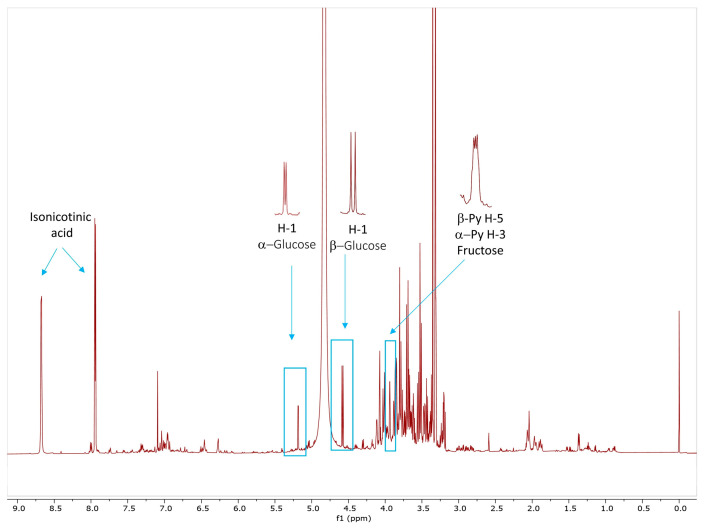
^1^H NMR spectrum of dry rose extract (DRE) in D_2_O/CD_3_OD (1:1) buffered with 1 M KH_2_PO_4_.

^1^H NMR spectroscopy has proven to be a valuable tool for the identification and quantification of primary and secondary metabolites of various plant extracts [23]. Preliminary examination of the dry rose extract (DRE) by ^1^H NMR showed the presence of a significant amount of carbohydrates (Figure 1). Two-dimensional NMR experiments and comparisons with literature data allowed unambiguous identification of glucose and fructose [24,25]. Thus, the anomeric proton resonances at 5.18 ppm (J = 3.8 Hz) and 4.58 ppm (J = 7.9 Hz) are diagnostic for α- and β-glucose. Fructose was identified by the signals at δ 4.07 (H-3 and H-4, β-furanose; H-3, α-furanose form) and δ 3.94 (H-5, β-pyranose and H-3, α-pyranose forms) [26].

Additionally, ^1^H NMR was used to quantify glucose and fructose in DRE using isonicotinic acid as an internal standard and the integral intensities of the selected diagnostic signals (Figure 1). The amount of glucose was obtained as the sum of the integration of the α- and the β-anomeric protons. Quantitation of fructose was performed using the signal at δ 3.94, corresponding to H-5 (β-pyr) and H-3 (α-pyr), considering the tautomeric equilibrium at this temperature as 65.76:26.38:5.21:2.39:0.26% of the different forms (β-pyranose, β-furanose, α-furanose, α-pyranose, and the keto forms) of fructose in D_2_O/CD_3_OD (1:1) buffered with 1 M KH_2_PO_4_, using an assignment for measured 2D spectra analogous to the methodology in [26]. Thus, the glucose and fructose contents in DRE were found to be 85 mg/g E and 108 mg/g E; i.e., the total sugar content calculated as the sum of glucose and fructose content was 193 mg/g E. The presence of fructose and glucose as well as galactose and sucrose has been recently reported in an extract obtained after industrial CO_2_ extraction of rose flowers [27]. Unfortunately, it is not possible to compare the results for these monosaccharides because the amount of glucose and fructose is only given as a percentage of TIC from GC-MS analysis.

#### 2.1.2. Identification of Compounds by HPLC-DAD-ESIMS, HPLC-HRMS and NMR

**Figure 2:**
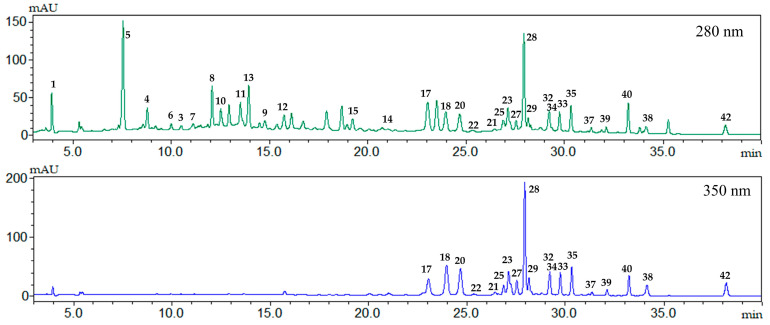
HPLC-DAD chromatogram of dry rose extract (DRE) at 280 (green) and 350 (blue) nm. For the peak identification see Table 1.

Dry rose extract (DRE) was investigated by HPLC-DAD-ESIMS and HPLC-HRMS (Table 1 and Figure 2). Thus, out of the 42 compounds, 25 were tentatively identified based on their chromatographic behavior parameters (UV absorption maxima, *m*/*z* values, molecular formula, and fragmentation pattern) and comparison with those described in the literature and open access LC-MS libraries. A total of 4 compounds were unambiguously identified as gallic acid, rutin, quercetin, and kaempferol with authentic standards, and the structure of 13 compounds was further confirmed by NMR (Tables S1 and S2, Figure S1). To achieve this, the DRE was re-extracted with ethyl acetate and the resulting EtOAc extract (EAE) was separated to give individual compounds. The identified compounds belong to two main metabolite classes, galloyl glycosides (gallotannins and ellagitannins) and flavonol glycosides, easily recognized by their characteristic UV absorption maxima at 280 and 340–360 nm (Table 1 and Figure 2).

Compound **1** had a deprotonated molecular ion [M − H]^−^ at *m*/*z* 191 and its MS/MS fragmentation gave a fragment ion at *m*/*z* 127, characteristic of quinic acid. Compounds **5**, **7,** and **17** showed a deprotonated molecular ion [M − H]^−^ at *m*/*z* 169, 153, and 301, respectively, and MS/MS fragmentation [M − H − 44]^−^ at *m*/*z* 125, 109, and 257, due to the neutral loss of a CO_2_ group, and were identified as gallic acid, protocatechuic acid, and ellagic acid, respectively. Compound **9** showed a deprotonated molecular ion at *m*/*z* 183 and an MS/MS fragment [M − H − 15]^−^ at *m*/*z* 168 corresponding to the loss of a CH_3_ group, suggesting that **9** is methyl gallate.

Four gallotannins (**2**, **4**, **6**, and **10**) were detected in DRE. Compound **2** showed a [M − H]^−^ at *m*/*z* 331, and characteristic fragment ions at *m*/*z* 169, corresponding to the presence of gallic acid; at *m*/*z* 125 ([M − H − 162 − 44]^−^) due to the subsequent decarboxylation of the gallic acid residue; and at *m*/*z* 271, corresponding to cross-ring cleavage of the hexose molecule (−60 Da). All these data supported the identification of **2** as galloyl hexose. Compounds **4** and **6** had the same [M − H]^−^ at *m*/*z* 483 and MS/MS fragmentation ions at *m*/*z* 331 resulting from the loss of a galloyl residue (−152 Da) and at *m*/*z* 169 due to the formation of a deprotonated gallic acid, consistent with digalloyl hexose isomers [19,28]. Compound **10** showed [M − H]^−^ at *m*/*z* 635, which in the MS/MS spectrum yielded fragments at *m*/*z* 465 and 313, representing the loss of a gallate unit (−170 Da) and subsequent loss of galloyl residue (−152 Da) as well as a fragment ion at *m*/*z* 169 corresponding to deprotonated gallic acid. Therefore, compound **10** was tentatively identified as a trigalloyl hexose.

Compounds **3**, **8**, **11**–**13**, **15**, and **30** belong to the group of ellagitannins as their MS/MS spectra contain a fragment at *m*/*z* 301, characteristic of ellagic acid. In addition, compounds **3** (*m*/*z* 633), **8**, **11**, and **13** (*m*/*z* 785), and **15** (*m*/*z* 937) displayed MS/MS fragments at *m*/*z* 275 due to the decarboxylation of a hexahydroxydiphenoyl (HHDP) moiety [28]. Comparison of their UV spectra and MS/MS fragmentation pathways with the literature data led to the tentative identification of these compounds as HHDP galloyl hexose (**3**) [28,29], HHDP digalloyl hexose (**8**, **11**, and **13**) [28,29], HHDP trigalloyl hexose (**15**) [28,29], flavogallonic acid (**12**), and flavogallonic acid methyl ester (**30**) [30]. Gallotannins and ellagitannins are common constituents of the plants of the Rosaceae family and have recently been reported in rose petals, distilled rose petals, and rose water [19,20,29].

Free aglycones, flavonoid mono- and di-glycosides, flavonoid-coumaroyl-glycosides and flavonoid-galloyl-glycosides were identified according to their UV behavior and mass spectral fragmentation [31], including 12 quercetin and 14 kaempferol derivatives (Table 1). Compounds **38** and **42** had [M − H]^−^ at *m*/*z* 301 and 285, respectively, and were identified as free aglycones quercetin and kaempferol by comparison of their UV, R_t_, and mass-spectral fragmentation with authentic standards.

Flavonol mono- and diglycosides were quercetin and kaempferol derivatives identified based on their abundant fragment ions appearing at *m*/*z* 301 for quercetin (**18**, **20, 21**, **26**, **27**, **29,** and **36**) and at *m*/*z* 285 for kaempferol (**23, 25**, **28, 32**–**35**, **39**, **40**, and **41**). The presence of a high-intensity radical aglycone ion at *m*/*z* 300 and 284 in their MS/MS spectra revealed a substitution at the 3-OH position in the structure of the quercetin and kaempferol derivatives, respectively [32]. In addition, the ^1^H NMR spectra of isolated compounds showed characteristic proton signals for 3,5,7,3′,4′-penta- (**18**, **20**, **26**, **29**, and **36**) and 3,5,7,4′-tetra- (**23**, **28**, **32**–**35**, **39**, and **40**) substituted flavones (Tables S1 and S2). The neutral loss of 162 Da from the precursor ion in the MS/MS spectra of **18**, **20**, **23,** and **28** revealed the presence of a hexose moiety. Furthermore, the observed differences in the multiplicity and the vicinal coupling constants of H-4″ in the ^1^H NMR spectra of **20** and **28** (δ 3.32–3.34, t, 9.0 Hz) and **18** and **23** (δ 3.82–3.84, brd, 3.2 Hz) identified these compounds as quercetin-3-*O*-β-glucopyranoside (isoquercitrin), kaempferol-3-*O*-β-glucopyranoside (astragalin), quercetin-3-*O*-β-galactopyranoside (hyperoside), and kaempferol-3-*O*-β-galactopyranoside, respectively. The neutral loss of 146 Da from the precursor ion in the MS/MS spectra, as well as the signal for the anomeric proton at δ 5.33/5.37 (d, 1.5 Hz) and for a methyl group at δ 0.93 (d, 6.5 Hz) in the ^1^H NMR spectra revealed the presence of an α-rhamnopyranosyl moiety in the structures of **29** and **35**. Therefore, compounds **39** and **35** were quercetin 3-*O*-α-rhamnopyranoside (quercitrin) and kaempferol 3-*O*-α-rhamnopyranoside (afzelin), respectively. The loss of 132 Da from the precursor ion in the MS/MS spectra of **32**, **26,** and **33** indicated the presence of a pentose moiety, which was determined to be β-xylopyranosyl and α-arabinofuranosyl from the multiplicities and the coupling constants of the anomeric protons in the ^1^H-NMR spectra of **32** (δ 5.17, 6.7 Hz), and **26** and **33** (δ 5.47, brs). Therefore, compounds **32**, **26**, and **33** were unambiguously identified as kaempferol-3-*O*-β-xylopyranoside, quercetin-3-*O*-α-arabinofuranoside (avicularin), and kaempferol-3-*O*-α-arabinofuranoside (juglanin). Compound **21** exhibited the same deprotonated molecular ion and fragmentation pattern in the MS/MS as **26** and was tentatively identified as quercetin-3-*O*-pentoside.

Compounds **25** and **34** (*m*/*z* 593 [M − H]^−^ and *m*/*z* 285), **39** (*m*/*z* 635 [M − H]^−^ and *m*/*z* 285), **36** (*m*/*z* 651 [M − H]^−^ and *m*/*z* 301), and **27** (*m*/*z* 609 [M − H]^−^ and *m*/*z* 301) were kaempferol and quercetin diglycosides. The ^1^H NMR spectra of **34**, **36,** and **39** clearly indicated the presence of glucopyranosyl (δ 4.48, d, 7.7 Hz) and rhamnopyranosyl (δ 5.35, d, 1.5 Hz) moieties and their connection was confirmed by COSY, HSQC, and HMBC experiments. An additional signal at δ 2.01 in the ^1^H-NMR spectra of **36** and **39** showed the signal of an acetyl group located at C-6″ of the glucopyranosyl part (δ 4.36 and 4.18, H-6a‴ and H-6b‴). Thus, compounds **34**, **39**, and **36** were identified as kaempferol-3-*O*-β-glucopyranosyl (1 → 4)-α-l-rhamnopyranoside (multiflorin B), kaempferol-3-*O*-[6‴-*O*-acetyl-β-d-glucopyranosyl] (1 → 4)-α-l-rhamnopyranoside (multiflorin A), and quercetin-3-*O*-[6‴-*O*-acetyl-β-d-glucopyranosyl] (1 → 4)-α-l-rhamnopyranoside, respectively. Compound **25** was tentatively determined as kaempferol-3-*O*-rutinoside by comparison of its mass-spectral data with the literature data [33], while compound **27** was identified as rutin by comparison of its UV, R_t_, and mass-spectral fragmentation with an authentic standard.

The UV spectra of compounds **37**, **40**, and **41** showed λ_max_ at 260 and 313 nm, suggesting that these flavonols were acylated [34]. Compound **40** was identified as kaempferol 3-*O*-(6‴-*O*-p-coumaroyl)-β-d-glucopyranoside (*trans*-tiliroside) as its MS/MS spectrum showed [M − H]^−^ at *m*/*z* 593 and a fragment ion at *m*/*z* 285 due to the elimination of a coumaroyl glucose unit (−308 Da) [35]. Furthermore, the ^1^H NMR spectrum contained characteristic proton signals for a *trans*-coumaroyl moiety whose position at C-6‴ was followed by the downfield shifts of the H-6″ signals of the glucopyranoside (Table S2). Compound **41** exhibited the same deprotonated molecular ion and MS/MS fragmentation pattern as **40** and was tentatively identified as *cis*-tiliroside. Compound **37** showed [M − H]^−^ at *m*/*z* 609 and MS/MS fragments at *m*/*z* 463 and 301 due to the subsequent elimination of p-coumaroyl and hexose moieties (−146 and 162 Da). In addition, the high-intensity ion at *m*/*z* 300 suggested that **37** was quercetin-3-*O*-p-coumaroyl hexoside.

Compounds **14**, **16**, and **22** were identified as quercetin-galloyl-hexoside as they exhibited [M − H]^−^ at *m*/*z* 615 and prominent fragments at *m*/*z* 463 and 301 due to the subsequent loss of 152 and 162 Da, which was indicative of a galloyl moiety and hexose. The presence of the galloyl group was also confirmed by the abundant peak at *m*/*z* 169 in their MS/MS spectra. Further, the high intensity of the radical aglycone ion at *m*/*z* 300 in the MS/MS spectra of peaks **14** and **16** suggested that they were quercetin-3-*O*-galloylhexoside. Similarly, compounds **19**, **24**, and **31** kaempferol-galloyl-hexosides showed [M − H]^−^ at *m*/*z* 599 and a fragment at *m*/*z* 285 corresponding to the loss of a galloyl hexose unit (−314 Da) in the MS/MS experiment. The intensive peak at *m*/*z* 284 in **19** and **24** identified these compounds as kaempferol-3-*O*-hexoside.

All identified compounds have been previously described in various extracts from fresh rose petals as well as from waste rose petals and water obtained after distillation [16,19,20,29,36,37,38]. It is worth noting that all these studies reported the presence of quercetin acetyldisaccharide, while the combined use of LC-MS/MS and ^1^H NMR in this work led to its unequivocal identification as quercetin 3-*O*-[6‴-*O*-acetyl-β-d-glucopyranosyl] (1 → 4)-α-l-rhamnopyranoside (**36**).

#### 2.1.3. Quantitative Analysis of Phenolic Compounds

The total phenolic (TPC) and total flavonoid (TFC) contents of the DRE and EAE, measured spectrophotometrically, were 212.19 ± 3.43 and 680.48 ± 2.48 mg GAE/g E and 135.28 ± 1.77 and 482.26 ± 1.82 mg RE/g E, respectively (Table S3). The results obtained for the TPC and TFC of DRE and EAE were significantly higher than those found for the methanol extract of the defatted flowers of fresh Taif rose (*R. damascena trigintipetala* Dieck) and fractions obtained after re-extraction of the methanol extract with EtOAc and *n*-butanol [17]. The authors in this study reported the highest TPC and TFC (343.19 mg GAE/g and 300.82 mg RE/g) for the ethyl acetate fraction whereas the *n*-butanol fraction and the crude methanol extract showed the lowest TPC and TFC (98.62 and 53.25 mg GAE/g and 53.91 and 31.27 mg RE/g, respectively). The TPCs of methanol extracts of fresh and spent flowers of *Rosa damascena* were 276.02 and 248.97 mg GAE/g, respectively [12]. In another study, cold methanolic extraction of fresh rose flowers yielded higher TPC and TFC (344.45 mg GAE/g and 56.81 mg RE/g) than hot methanolic extraction (233.56 mg GAE/g and 50.04 mg RE/g [15]. TPC and TFC in the aqueous residue of rose hydrodistillation and in the enriched polyphenol extract obtained by purification with macroporous resin polystyrene-FPX66 were 170 and 260 mg GAE/g and 24.7 and 80 mg QUE/g, respectively [36].

The results obtained for the content of individual compounds (Table 1) by HPLC-DAD revealed that flavonoids were the predominant class of phenolic compounds in DRE and EAE. The total amounts of flavonoids were 109.24 and 457.27 mg HypE/g E (~68% of all quantified compounds in DRE and EAE). Flavonoid content was four times higher in EAE compared to DRE. The re-extraction with ethyl acetate appears to give better results than the XAD 16 HP purification of the 30% aq. ethanol extract of distilled rose petals, which increased the total yield of flavonoids only twofold [38]. Enzyme-assisted extraction of rose petals was recently reported as an approach to increase the yield of the individual flavonols by 1.5–1.8-fold [29]. Kaempferol glycosides accounted for 43% of the total compounds that were quantified for both DRE and EAE, with kaempferol 3-*O*-glucoside being the predominant compound (15.7 and 16.4%). Quercetin glycosides were ~25% of the total quantified compounds in both DRE and EAE. Hyperoside (7.6 and 7.2%) and isoquercitrin (6.7 and 6.3%) were found to be the major quercetin derivatives. The predominance of kaempferol glycosides in the studied DRE is consistent with other studies on rose flowers [16,29,38,39]. However, there are some investigations that report the predominance of quercetin glycosides in the rose petal extracts [17,19,29]. These differences can be explained by the different origins and/or extraction processes of the plant material. The total amounts of hydrolysable tannins (gallic acid, ellagic acid, gallotannins, and ellagitannins) in DRE and EAE were 48.486 and 211.77 mg GAE/g E (~31% of all quantified compounds) with gallic and ellagic acid being the main components. Although the presence of hydrolysable tannins in various rose extracts has been reported, there is a lack of information on the contents of individual compounds to compare with our results.

### 2.2. Antioxidant Potential

Antioxidant assays (DPPH, ABTS, and FRAP) based on different mechanisms were applied to investigate the antioxidant capacity of DRE and EAE. The DPPH scavenging assay is widely used for preliminary evaluation of the antioxidant potential of extracts and is based on the donation of electrons by the antioxidants to neutralize the DPPH radicals [40]. The EAE demonstrated higher DPPH radical scavenging activity (IC_50_ 0.16 ± 0.01 mg/mL) in contrast to that obtained from DRE (IC_50_ 0.27 ± 0.01 mg/mL) (Table 2). Comparison of the observed IC_50_ values with those of the commercial antioxidant BHT and caffeic acid showed that both extracts were better scavengers of DPPH radicals than BHT and weaker antioxidants than caffeic acid. The ABTS assay is another method to determine the antiradical scavenging ability based on the hydrogen atom donating tendency of phenolic compounds [40]. The results obtained in the ABTS assay were similar to those of the DPPH assay (Table 2). The EAE showed 1.8 times higher antioxidant capacity compared to DRE. The FRAP assay is a typical single-electron-transfer-based method measuring the reduction of the ferric ion (Fe^3+^)–ligand complex to an intense blue ferrous complex (Fe^2+^) using antioxidants in an acidic environment [40]. EAE showed a reducing power similar to that of caffeic acid and better activity than that obtained from DRE (Table 2). It is worth mentioning that despite the higher amounts of TPC, TFC (Table S3), and individual compounds in EAE compared to DRE (3–4 times), the antioxidant activity measured by the three methods in EAE was only 0.5–2 times higher than that in DRE. This result could be explained by the different contributions of the individual compounds to the antioxidant potential of the extracts as well as to their potential mutual interactions which can be synergistic, antagonistic, or additive [41,42,43,44].

The antioxidant activities, including DPPH, ABTS, and FRAP, of different rose extracts have been already reported [12,15,17,18,19,36], but it is difficult to compare the results due to differences in the assay procedures, or in the solvents used for extraction. Thus, the methanol extract of defatted fresh Taif rose exhibited DPPH radical scavenging activity with a SC_50_ of 49.44 µg/mL [17]. The ethyl acetate fraction obtained from the sequential fractionation of the same extract showed higher radical scavenging activity than the *n*-butanol fraction (SC_50_ 15.62 and 36.29 µg/mL, respectively) [17]. In another study, the DPPH antiradical activity of methanol extracts was changed from 65.88% in the hot extraction of spent flower at 50 µg/mL to 89.86% in the cold extraction of fresh flower at 150 µg/mL and was comparable to that of the common antioxidants BHA and BHT [15]. Similarly, methanol extracts from fresh and spent rose flowers showed 74.51 and 75.94% inhibition of DPPH radical activities at 100 ppm [12]. The DPPH and ABTS radical scavenging activity of different ethanolic extracts of waste rose flowers ranged from 951.7 to 1448.7 µM Trolox/g DW and from 1175.1 to 1548.0 µM Trolox/g DW, respectively [18]. Recently, strong DPPH radical scavenging activity (39,138.90 μM TroloxE/100 g) and high ferric ion reducing antioxidant power (FRAP, 35,550 μM TE/100 g) were reported for dry-pressed distilled rose petals [19]. The extract prepared from rose water after passing through macroporous resin displayed DPPH and ABTS inhibition with IC_50_ values of 25.4 and 8.7 μg/mL, respectively [36]. Antioxidant properties measured by the FRAP assay for fresh and spent flowers were 0.61–0.65 and 0.81–0.85 μg/mL at the concentrations of 100 and 150 μg/mL [15].

### 2.3. Antimicrobial Activity

**Figure 3:**
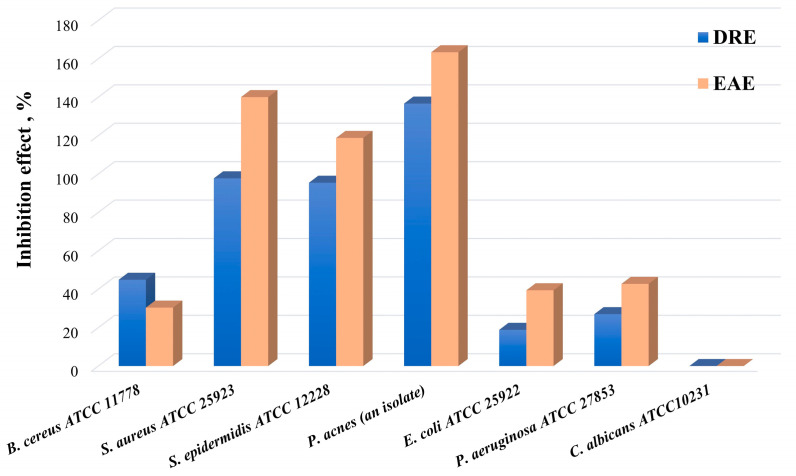
Inhibition effect of DRE and EAE on test pathogens.

The results of the antimicrobial activity tests of two extracts (DRE and EAE) have shown well-expressed activity against the different bacterial test pathogens (Table 3 and Figure 3). No activity was detected against the fungal test pathogen *Candida albicans*. Higher inhibitory activity was determined against *Propionibacterium acnes*, *Staphylococcus aureus*, and *Staphylococcus epidermidis* with an inhibitory effect of over 100% (Figure 3). The activity of EAE was more pronounced in five of the tested pathogens.

The minimum inhibitory concentrations (MICs) of the tested extracts were determined by an agar microdilution technique, which allows overcoming the solubility problems of plant extracts and measuring the growth of the test pathogens in a colored and highly opaque medium [45]. The MICs (mg/mL) of two extracts for each test pathogen are presented in Table 4. Regarding the EAE, the MICs for the pathogens used ranged between 2.5 and 10 mg/mL, while for DRE the MICs were higher for five of them.

Many authors have studied the antimicrobial activity of different types of extracts and products obtained during the processing of rose oil. Methanol extracts from rose flowers have been reported to exhibit a wide spectrum of antibacterial activity [12]. Maruyama et al. [10] highlighted in their research the strong bactericidal effect of rose water against *Staphylococcus aureus* (MRSA). The investigation of the antimicrobial effect of rose oil distillation wastewater showed the inhibition of *S. aureus* proliferation [9]. Denkova et al. [7] have determined the highest antimicrobial activity of 70% for hydroalcoholic extracts of rose waste materials, which inhibited the growth of the test pathogenic bacteria and yeast to varying degrees, with MICs ranging from 6 ppm to 600 ppm. Pires et al. [11] determined the MICs of hydromethanol extracts of rose flowers against various test pathogens, including *Escherichia coli*, *Pseudomonas aeruginosa*, and *Staphylococcus aureus*, falling within the range between 0.625 and 20 mg/mL. The obtained MICs for the two examined extracts (DRE and EAE) correspond to those reported by the cited authors.

Kaempferol and quercetin glycosides as well as the hydrolysable tannins (gallotannins and ellagitannins) have been shown to possess antimicrobial activity, especially against *E. coli* and *S. aureus* [39,46,47]. Therefore, they contributed to the broad spectrum of antibacterial activity observed in the DRE and EAE studied.

### 2.4. Cytotoxicity and Cell Cycle Alterations of Human Skin Fibroblasts and Human HepG2 Hepatocarcinoma Cells after Treatment with DRE and EAE

The utilization of plant components in biomedicine and cosmetics requires that they exhibit low toxicity to normal diploid human cells and do not significantly affect the cell cycle of differentiated cells. Crystal violet staining was employed to evaluate the cytotoxicity, as this method offers an estimate of the total amount of cells remaining after treatment (cell survival) and remains unaffected by the presence of polyphenols in the medium, unlike some enzyme activity assays.

**Figure 4:**
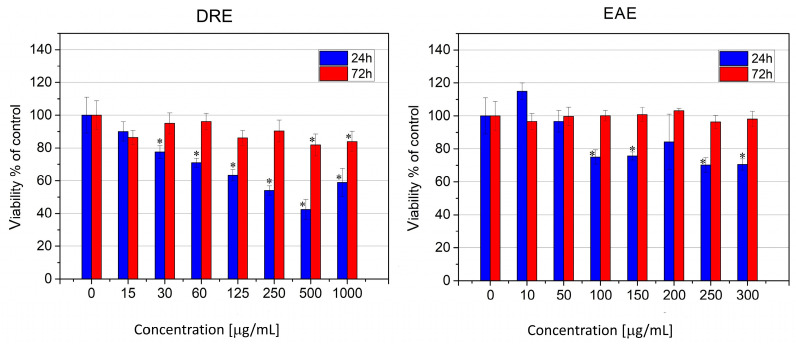
Cytotoxicity of rose extracts tested at 24 (blue bars) and 72 (red bars) hours after treatment of human skin fibroblasts. Data are presented as mean ± SD. *—Statistically different data (*p* < 0.05).

The tests performed showed low toxicity of both types of extracts to normal human fibroblasts (Figure 4). At the initial stage of treatment (at 24 h), a decrease in cell survival was observed for both extracts, albeit more pronounced for DRE, where a distinct dose dependence was also observed. Values corresponding to the IC_50_ for cell survival were reached only with DRE, at a concentration of 375 µg/mL (Figure 4). EAE had a less toxic effect on normal human fibroblasts; at concentrations above 100 µg/mL and up to 300 µg/mL, cell survival was about 70%, and this value did not change statistically significantly with increases in dosage. At the lowest concentration tested (10 µg/mL), an increase in the staining signal was observed, which could be due either to an increased number of cells or to increased metabolic activity and protein synthesis because of the impact of flavonoids (Figure 4).

In both examined extracts, recovery of cell viability was observed after 72 h of treatment, even at high concentrations. Therefore, it can be assumed that the cellular changes that occurred as a result of the treatment are reversible and that normal human fibroblasts fully recover from the initial stress.

**Figure 5:**
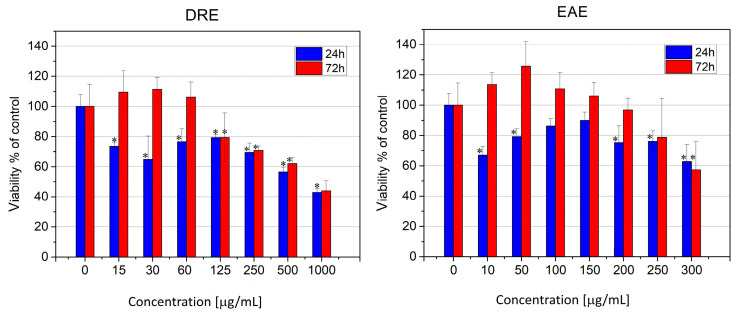
Cytotoxicity of *Rosa damascena* extracts on HepG2 human hepatocarcinoma cells after 24 (blue) and 72 (red) hours of treatment. Data are presented as mean ± SD. *—Statistically different data (*p* < 0.05).

The effect on human hepatocarcinoma (HepG2) cells was quite different (Figure 5). DRE at concentrations up to 60 µg/mL showed weak toxicity at 24 h, which was fully compensated for by 72 h, while concentrations above 650 µg/mL showed pronounced cytotoxicity, proportional to the extract concentration up to 1000 µg/mL, which was not compensated for on the third day of treatment (Figure 5).

EAE also showed low toxicity in these cells and did not reach IC50 in the concentration range studied. In contrast to normal cells, in this case, treatment with a low concentration of 10 µg/mL caused a loss of viability in about 35% of the cells, but at 72 h of recovery, an increase in signal above that of control untreated cells was again observed. In contrast to DRE, EAE at concentrations above 350 µg/mL was cytotoxic and reached IC50 values only after 72 h of treatment.

**Figure 6:**
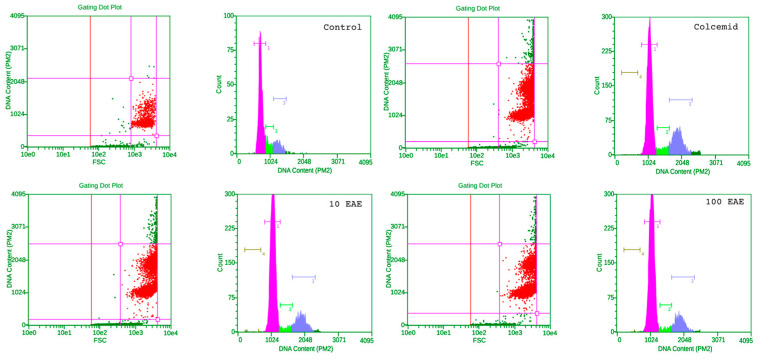
Flow cytometry of HSF cells treated with a low dose (10 µg/mL) and the first effective dose of 100 µg/mL of EAE. Dot plots and histograms of representative experiments are shown. Cell populations included in the analysis are represented by red dots. In the histograms, pink represents the count of cells in G0/G1 phase, green the count of cells in S phase and purple the count of cell in G2/M phase of the cell cycle.

**Figure 7:**
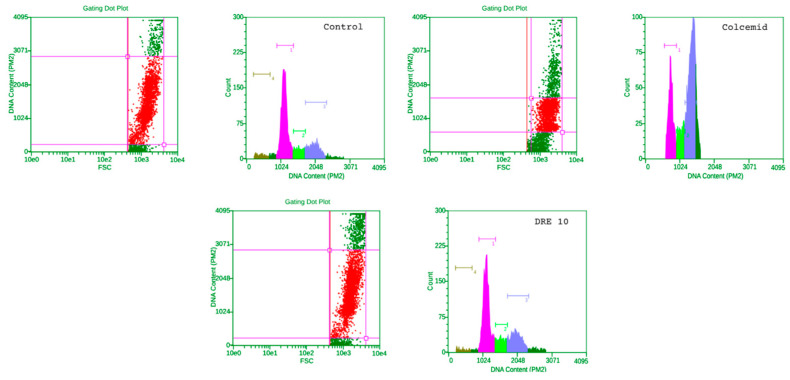
Flow cytometry of HepG2 cells treated with a low dose (10 µg/mL) of DRE. Dot plots and histograms of representative experiments are shown. Cell populations included in the analysis are represented by red dots. In the histograms, pink represents the count of cells in G0/G1 phase, green the count of cells in S phase and purple the count of cell in G2/M phase of the cell cycle.

The increased signal of CV-assay upon long-term treatment (72 h) with low concentrations in both cell types is probably due to the activation of proliferation. This was the reason for performing a flow cytometric analysis of the cell cycle on the third day of treatment of the two cell types studied (Figure 6 and Figure 7). Normal human diploid fibroblasts responded relatively weakly to colcemid, which was used as a positive control to block the cell cycle in G2, as a result of microtubule disruption (Figure 6, upper right panel). The cell population in G2 as a result of the effect of colcemid increased by about 10.3% at the expense of a reduced number of cells in the G1 (by 5.5%) and S periods (by 5.5%).

Conversely, after treatment with EAE, there was an increase in the population in G1 of 3% at the lowest concentration and 6% at the highest concentration, a decrease in the number of cells in replication of about 6% for both concentrations tested and a slight increase in the arrested population in G2—4% at the lowest concentration and 1% at the highest concentration, respectively. These changes in the distribution of cells in the phases of the cell cycle are most likely due to reversible stress induced by the effects of plant metabolites, which causes a slight arrest of the cycle at the main checkpoints in the pre- and post-synthetic period, and an increase in metabolic activity, to overcome which we report an increased signal in the crystal violet test.

Cells of tumor origin (HepG2) were significantly more sensitive to agents blocking microtubule polymerization, as also seen in our results (Figure 7, upper right panel). Cells in G1 decreased by 20%, at the expense of an increase in the population in G2 by 33% and those in S by about 5%. Treatment with DRE and EAE did not change the distribution of cells and the groups were almost identical to the control untreated cells, with a slight increase in their amount in G1.

Recently, the methanol extract of *Rosa damascena* Mill var. *trigentipetala* from Saudi Arabia showed a good anticancer effect on HepG2 cells by causing cell cycle arrest in G2 and induction of programmed cell death after 48 h treatment with an IC50 range of 100–150 µg/mL [48]. Anticancer effect on Caco-2 human colon carcinoma with an IC_50_ of 180 µg/mL has been found for *Rosa damascena* Mill callus extracts [49]. Another group (from Turkey) reported the anticancer effect of methanol extract from *R. damascena* on HeLa cells with an IC_50_ of 265 µg/mL [50]. The extracts in our studies did not cause such deviations in growth characteristics and IC_50_ was found only for the DRE at a much higher concentration (above 650 µg/mL). Abnormalities in cell growth were observed at the lower concentrations of the extract, and we checked the distribution of HepG2 cells in the cell cycle phases at the lowest concentration, but no changes in distribution and signs of apoptosis were observed. The cell cycle of normal HSF also showed minor changes. This can be explained by the different composition of metabolites from plants belonging to different varieties and grown in different climatic conditions. Similar results were also reported by Georgieva et al. [20] when studying several *Rosa* species: *R. damascena* Mill, *R. alba* L., *R. gallica* L., and *R. centifolia* L. grown in Bulgaria. None of the tested extracts from the four rose species exerted significant cytotoxic effects on the selected human cancer and normal cell lines. The absence of apoptosis in HepG2 cells treated with *R. damascena* Mill extract was also confirmed by these authors [20]. Low toxicity and beneficial effects on wound healing in a diabetic rat model of a combination of retinoic acid and hydroalcoholic rose extract were also reported [51].

## 3. Materials and Methods

### 3.1. Rosa damascena Mill Sample

Dry rose extract (DRE) was provided by Galen N Ltd. (Bulgaria). Dry rose extract (DRE) is produced in multi-kilogram quantities by the company Galen N Ltd. (Bulgaria) and was provided as a gift for the purposes of the present research. The specifications of the standardized extract are publicly available (https://galen-n.com/bulgarian-rose-dry-extract/ (accessed on 14 November 2023)). The rose blossoms (flowers) of *Rosa damascena Mill* (Damask rose) are collected from the company’s plantation in the village of Zelenikovo in the so-called Rose Valley of Bulgaria. Petal extraction was performed with aqueous ethanol (water/ethanol = 70:30) at a dry material-to-solvent weight ratio of 1:10. Dry rose extract is obtained after concentration under vacuum and spray drying. Dry rose extract is a fine powder of pale brown to reddish color. 

### 3.2. Extraction and Isolation of Individual Compounds

The dry rose extract (1 g) was dissolved in water (20 mL) and extracted with EtOAc (3 × 20 mL). The combined EtOAc extracts were evaporated under reduced pressure to obtain the EtOAc extract, EAE (180 mg). A portion of the EAE (100 mg) was subjected to MPLC on LiChroprep RP-8 and eluted with increasing concentrations of CH_3_OH in H_2_O (20 to 80%) to yield 10 fractions. Fr. 1 (15.6 mg) contained hyperoside (**18**) and isoquercitrin (**20**) in a ratio of 1:1 (as deduced by NMR). Prep. TLC (CHCl_3_/CH_3_OH/H_2_O, 60:15:4) of fr. 2 (2.5 mg) afforded avicularin (**26**) (1.3 mg). Prep. TLC (CHCl_3_/CH_3_OH/H_2_O, 60:15:4) of fr. 4 (20.9 mg) yielded astragalin (**23**) (2.1 mg), a mixture of astragalin (**23**) and quercitrin (**29**) (15.5 mg, ratio 1:0.2), and kaempferol 3-*O*-β-d-galactopyranoside (**27**) (1.2 mg). Prep. TLC (CHCl_3_/CH_3_OH/H_2_O, 60:15:4) of fr. 6 (7.5 mg) afforded kaempferol 3-*O*-α-l-arabinofuranoside (**33**) (1.4 mg), kaempferol 3-*O*-xylopyranoside (**32**) (1.2 mg), and multiflorin A (**34**) (1.0 mg). Prep. TLC (CHCl_3_/CH_3_OH/H_2_O, 60:15:4) of fr. 7 (4.1 mg) yielded kaempferol 3-*O*-α-l-rhamnopyranoside (**35**) (1.6 mg) and quercetin 3-*O*-[6-*O*-acetyl-β-d-glucopyranosyl] (1→4)-α-l-rhamnopyranoside (**36**) (0.8 mg). Prep. TLC (CHCl_3_/CH_3_OH/H_2_O, 60:15:4) of fr. 9 (4.9 mg) afforded trans-tiliroside (**40**) (1.2 mg) and multifolrin B (**39**) (1.1 mg). The structures of the isolated compounds were determined by comparison of their ^1^H NMR spectral data (CD_3_OD) with those reported in the literature [37,52,53,54,55,56].

### 3.3. Qualitative and Quantitative NMR Analysis of Monosaccharides

The ^1^H NMR spectra were recorded on a Bruker Avance NEO 600 spectrometer (Biospin GmbH, Rheinstetten, Germany) at 298.0 ± 0.1 K. ^1^H spectra in CD_3_OD/D_2_O with 1 M KH_2_PO_4_ buffer (*zg30* pulse sequence) were acquired using 64 scans, 64K data points, acquisition time of 4.19 s, and relaxation delay of 30 s. Assignment of the proton signals for the different tautomeric isomers of fructose and their quantitation was performed by the acquisition of 2D NMR spectra of fructose in the buffered CD_3_OD/D_2_O solution using pulse sequences from the standard Bruker library (*cosygpmfqf*, *roesyphpr.2*, *hsqcedetgpsp.3*, *hmbcgplpndqf*) analogous to the methodology in [26].

For quantitative analysis, the extract (10 mg) was dissolved in a mixture (1:1) of CD_3_OD and 1 M KH_2_PO_4_ buffer in D_2_O (pH 6.0) containing 0.01% sodium trimethylsilyl propionate (TSP-d_4_) and isonicotinic acid at a concentration of 2 mg/mL as standard (0.5 mL). Baseline correction and integration were performed manually. The amounts of compounds in the studied mixtures were based on the integral intensities of the respective signals for the individual compounds without overlapping. The two-proton multiplet at δ 8.68 of *iso*-nicotinic acid was used as an internal standard. Quantitation was performed using the following general Equation (1):

m_x_ = (m_s_ × N_s_ × I_x_ × M_x_)/(I_s_ × M_s_ × N_x_) (1)

where m_x_ is the mass of the compound being measured, m_s_ is the weighted mass of the standard; M_s_ and M_x_, I_s_ and I_x_, and N_s_ and N_x_ are the molar masses (in Da), the integrated signal area, and the number of protons for the corresponding integrated signal of the standard and the compound, respectively. The contents of the individual compounds were expressed as mg/g E (extract).

### 3.4. HPLC-HRMS Analysis

Analyses were carried out on Vanquish UHPLC systems coupled with a Q Exactive Plus Hybrid Quadrupole-Orbitrap Mass Spectrometer (Thermo Fisher Scientific, Bremen, Germany), following a modified procedure from the literature (cit). HPLC separations were performed on a Restek, Raptor C18, (2.7 µm, 150 × 2.1 mm) equipped with a Guard Column Cartridge Restek, Raptor C18 EXP (2.7 µm, 5 × 2.1 mm) at 40 °C. The mobile phase consisted of water containing 0.1% (*v*/*v*) formic acid (A) and acetonitrile (B). The following gradient program was performed: 0–25 min, 5–30% B; 25–27 min, 30–50% B; 27–29 min, 50–95% B; 29–34 min, 95% B; 34–35 min, 5% B; 35–40 min, 5% B. The flow rate was 0.3 mL/min and the sample injection volume was 1 µL. The operating conditions for the HESI source were as follows: −3.75 kV spray voltage; 300 °C capillary temperature; sheath gas flow rate, 30 arb. units; auxiliary gas flow, 7 arb. units; S-Lens RF level, 50 V. Nitrogen was used as nebulizing gas and as the collision gas in HCD cells. Full-scan mass spectra over the range 120–1200 were acquired in negative ionization mode at resolution settings of 70,000, automatic gain control (AGC) target of 1e6, and a maximum injection time (IT) of 80 ms. Top5 mode of operation was used for qualification of the compounds, where ddMS^2^ conditions were set to resolution 17,500, AGC target 5e5, max. IT 50 ms, isolation window 2.0 *m*/*z*, and stepped normalized collision energy (NCE) of 20, 40, and 70. Data acquisition and processing were carried out using Xcalibur software version 4.2 SP1 (Thermo Fisher Scientific) and FreeStyle program version 1.5 (Thermo Fisher Scientific).

### 3.5. Qualitative and Quantitative HPLC-DAD-ESI/MS Analysis

HPLC-DAD-ESI/MS analysis of DRE and EAE was performed on a Shimadzu LC-2040C 3D Nexera-I and Shimadzu LCMS 2020 (single quadrupole), using column Force C18 (Restek, Bellefonte, PA, USA), 150 mm × 4.6 mm, 3 µm, thermostated at 40 °C. The UV spectra were recorded from 190 to 800 nm. The ion spray voltage was set in the negative mode at −4.50 kV; scan range: 100–1000 *m*/*z*; interface temperature: 350 °C; desolvation line: 250 °C; heat block: 200 °C; nebulizing gas flow: 1.5 L/min; and drying gas flow: 15 L/min. The mobile phase consisted of water containing 0.1% (*v*/*v*) formic acid (A) and acetonitrile (B). The following gradient program was performed: 0–7 min, 5–15% B; 7–20 min, 15–20% B; 20–25 min, 20–30% B; 25–40 min, 30–50% B; 40–42 min, 95% B; 42–47 min, 95% B; 47–48 min, 5% B; 48–53 min, 5% B. The flow rate was 0.4 mL/min, and the injected volume was 5 µL.

Two phenolic standards (gallic acid and hyperoside) were employed for the quantification of the phenolic content in the two extracts. Gallic acid (1, 10, 25, 50, and 100) mg/L, y = 35,223x − 25,342; r^2^ = 0.999; hyperoside (1, 10, 25, 50, and 100) mg/L, y = 35,223x − 25,342; r^2^ = 0.998. DRE was dissolved in methanol/H_2_O at a concentration of 2000 mg/L, while EAE was dissolved in MeOH at a concentration of 500 mg/L. Quercetin and kaempferol glucosides were quantified as mg hyperoside equivalents per gram extract (mg HypE/gE) at 350 nm, while gallic and ellagic acids and galloyl glucosides were quantified as mg gallic acid equivalents per gram extract (mg GAE/gE) at 280 nm. All measurements were performed in triplicate.

### 3.6. Determination of Total Phenolic and Total Flavonoid Content

Total phenolic content (TPC) was measured using the Folin–Ciocalteu method [57]. The concentration was calculated using gallic acid as a standard and the results were expressed as milligrams (mg) of gallic acid equivalents (GAE) per gram of extract (mgGAE/gE). Total flavonoid content (TFC) was measured using a previously developed colorimetric assay [58]. Concentration was calculated using a rutin calibration curve and the results were expressed as milligrams of rutin equivalents per gram extract (mgRE/gE). All measurements were performed in triplicate.

### 3.7. Determination of Antioxidant Capacity

#### 3.7.1. DPPH Radical Scavenging Activity

The 1,1-diphenyl-2-picrylhydrazyl radical (DPPH) scavenging activity assay was performed according to the procedure described by Thaipong et al. [59]. The IC_50_ values were obtained by the plotting DPPH scavenging percentage of each sample versus concentration. Butylated hydroxytoluene (BHT) and caffeic acid were used as standards for comparison of antioxidant potential. All measurements were performed in triplicate.

#### 3.7.2. ABTS Radical-Ion Scavenging Activity

ABTS (2,2′-azinobis 3-ethylbenzothiazoline-6-sulfonic acid) radical-ion scavenging activity was performed according to the procedure previously described by Thaipong et al. [59]. The results are expressed as Trolox equivalents of antioxidant capacity (mM Trolox equivalents per gram extract), using a calibration curve of different concentrations of Trolox in methanol (100–500 µM). All measurements were performed in triplicate.

#### 3.7.3. FRAP Activity

The assay was performed according to Benzie and Devaki with slight modifications [60]. The FRAP reagent was freshly prepared by mixing 10 parts of 0.3 M acetate buffer (pH 3.6), 1 part of 2,4,6-tri(2-pyridyl)-1,3,5-triazine (TPTZ) in 40 mM HCl, and 1 part of 20 mM FeCl_3_·6H_2_O in distilled H_2_O. The reaction was started by mixing 3 mL FRAP reagent with 100 μL of the investigated sample (diluted with MeOH if necessary). The reaction time was 30 min at room temperature in the dark and the absorbance was measured at 593 nm against a blank. The FRAP value was calculated from a calibration curve of FeSO_4_·7H_2_O standard solutions and expressed as mM Fe^2+^/g E. Butylated hydroxytoluene (BHT) and caffeic acid were used as standards for comparison of antioxidant potential. All measurements were performed in triplicate.

### 3.8. Antimicrobial Activity

Antimicrobial activity was determined by the disc diffusion method using two described samples with a concentration of 8 mg/disk per sample. Six bacterial test pathogens, *Bacillus cereus* ATCC 11778, *Escherichia coli* ATCC 25922, *Staphylococcus aureus* ATCC 25923, *Staphylococcus epidermidis* ATCC 12228, *Propionibacterium acnes* (an isolate), *Pseudomonas aeruginosa* ATCC 27853, and pathogenic yeast *Candida albicans* ATCC 18204 were used to screen for antimicrobial activity of samples. Overlays of test pathogens (0.5 McFarland) were prepared on agar plates. An amount of 30 µL of a pre-prepared solution of each sample was added to the sterile disks to achieve a working concentration and allowed to diffuse. Control disks with 30 µL 5% DMSO were used. The plates were incubated at the respective temperatures for each test pathogen of 37 °C and 30 °C for 24 h. Clear zones around the disk confirmed the antimicrobial activity and the inhibition zone diameters were measured in millimeters. The percentage of inhibition effect was calculated as follows: the percent of inhibition effect = (diameter of clear zone of sample/diameter of clear zone of the positive control) × 100.

Minimal inhibitory concentrations were determined using the agar microdilution method according to [45], with modifications. The extracts were dissolved in 5% DMSO and different concentrations were then prepared with molten Mueller–Hinton agar, vortexed, and placed in a water bath at 50–55 °C. An amount of 100 µL per well of each concentration was dispensed into 96-well microplates. Overnight cultures of pathogens were standardized to 0.5 McFarland (10^8^ CFU/mL) and then diluted to 10^7^ CFU/mL using saline solution. An amount of 3 µL from each standardized culture was dropped into the respective wells and the microplates were incubated for 24 h at the optimal temperature for the pathogens. In the same microplate, the uninoculated negative control and the positive growth control were carried out for each strain tested.

### 3.9. Cytotoxicity Tests

The cytotoxicity of the extracts was evaluated by the crystal violet assay performed on human diploid fibroblasts (HSF) and human hepatocarcinoma cell line HepG2. Cells were cultivated for 24 h in a 96-well flat-bottomed plate at a starting concentration of 2 × 10^4^ cells per well. The treatment was performed for 24 and 72 h with extracts diluted in DMEM at a concentration ranging from 0 to 1000 µg/mL for DRE and 0–300 µg/mL for EAE. Untreated cells were used as controls. The test followed the Cold Spring Harbor Laboratory protocol, as described in [61]. The absorbance was measured at 570 nm using an Epoch Microplate Spectrophotometer, BioTek® Instruments Inc., Winooski, VT, 05404-0998, USA with the Gen5^TM^ Data Analysis software, version 1.11.5. Data are presented as a percentage of the mean control value (the absorbance of untreated cells).

Cell cycle analysis was performed using a Guava easy Cyte flow cytometry system and Guava^®^ Cell Cycle Reagent, Luminex. Briefly, cells were treated with Rosa extracts at a low (10 µg/mL) and the first effective dose of 100 µg/mL for 72 h. Untreated cells (negative) and cells treated with 0.1 µg/mL colcemid for 4 h (positive) were used as controls. After treatment, the cells were washed with sterile PBS, trypsinized, and fixed with ice-cold 70% ethanol in Eppendorf tubes. Cells were stained as described by the manufacturer and the cell cycle data of each sample were acquired on the Guava easy Cyte instrument. The distribution of cells in the cell cycle phases was performed depending on the DNA content in each cell.

### 3.10. Statistical Analysis

All experiments were performed in triplicate. Results are presented as the mean ± standard deviation (SD). One-way ANOVA was performed for statistical analysis, followed by Tukey’s post hoc test, to detect differences between controls and samples when testing for antimicrobial activity. A *p*-value of *p* < 0.05 was considered as a statistically significant difference. For the cytotoxicity assay, statistical analysis was performed by one-way ANOVA using the Origin Pro 9.0 software at a significance level of *p* < 0.01 followed by Tuckey’s and Dunnett’s tests to statistically evaluate the differences between individual experimental groups.

## 4. Conclusions

In summary, the current study sheds light on the chemical constituents and biological properties of industrially produced dry rose extract obtained by aqueous ethanol extraction from fresh flowers of R. damascena. The extract exhibits strong antioxidant and well-expressed antibacterial activity, particularly effective against Propionibacterium acnes, Staphylococcus aureus, and S. epidermidis. These remarkable bioactivities are due to the abundance of various phenolic compounds. Specifically, the presence of phenolic acids and their esters, galloyl glycosides, ellagitannins, as well as kaempferol and quercetin glycosides collectively contribute to these effects. Kaempferol glycosides were the main class of compounds, with kaempferol-3-glucoside as the main compound. In addition, dry rose extract contained glucose and fructose.

Furthermore, the phenolic-enriched fraction obtained by re-extracting the dry rose extract with EtOAc showed an identical chemical profile but higher amounts of TPC, TFC, and individual compounds. Therefore, this enriched fraction demonstrated increased antioxidant and antibacterial activity against the tested pathogens. Both extracts showed low toxicity to normal human skin fibroblasts, while at relatively high concentrations, they exhibited toxicity towards human hepatocarcinoma HepG2 cells. At lower concentrations, the extracts were non-toxic and did not significantly affect the cell cycle parameters of either of the cell lines. Based on our comprehensive investigations, it is evident that both dry rose extract and its phenolic-enriched fraction hold considerable promise as sources of bioactive compounds. In conclusion, the remarkable bioactive properties of these extracts represent a promising avenue for innovative applications in the cosmetic, food, and pharmaceutical industries.

## Figures and Tables

**Table 1:** Identification of phenolic compounds by HPLC-DAD-ESIMS and HPLC-HRMS/MS and their content in DRE and EAE.

No	Rt ^a^ (min)	Rt ^b^ (min)	Compound	UV ^a^, λ_max, nm_	[M − H]^− b^ *m*/*z*	Δ ^b^, ppm	Formula ^b^	MS/MS Fragments ^b^	DRE ^c^	EAE ^c^
1	0.94	3.56	Qunic acid	-	191.0552	1.08	C_7_H_11_O_6_	**191**, 127, 85	nq	nq
2	0.98	-	Galloyl hexose	-	331.0674	1.00	C_13_H_15_O_10_	331, 271, **169**, 125	nq	nd/nq
3	1.01	10.56	HHDP galloyl hexose	285.6	633.0737	0.63	C_27_H_21_O_18_	633, 463, **301**, 275, 249, 231, 169	0.867 ± 0.002	2.051 ± 0.002
4	1.46	8.84	Digalloyl hexose	276.8	483.0783	0.46	C_20_H_19_O_14_	483, 331, **169**, 125	2.847 ± 0.095	4.633 ± 0.006
5 *	1.47	7.63	Gallic acid	271.8	169.0132	0.29	C_7_H_5_O_5_	169, **125**	13.402 ± 0.025	54.318 ± 0.092
6	1.92	10.06	Digalloyl hexose	281.8	483.0784	0.83	C_20_H_19_O_14_	483, 331, **169**, 125	1.217 ± 0	2.537 ± 0.004
7	2.58	11.2	Protocatechunic acid	259.2, 294.4	153.0182	−0.11	C_7_H_5_O_4_	153, **109**	1.006 ± 0.015	4.069 ± 0.051
8	4.45	12.12	HHDP digalloyl hexose	270.5	785.0849	−0.73	C_34_H_25_O_22_	785, 483, **301**, 275, 249, 169, 125	4.907 ± 0.006	11.884 ± 0.042
9	4.52	14.82	Methyl gallate	271.8	183.0290	1.13	C_8_H_7_O_5_	**183**, 168, 137, 124	1.660 ± 0.006	7.907 ± 0.010
10	5.08	12.56	Trigalloyl hexose	278.0	635.0899	1.54	C_27_H_23_O_18_	635, 465, 412, 313, **169**, 125	1.962 ± 0.034	7.744 ± 0.030
11	6.37	13.54	HHDP digalloyl hexose	13.54	785.0853	1.32	C_34_H_25_O_22_	785, 483, **301**, 275, 249, 169, 125	3.277 ± 0.015	11.454 ± 0.264
12	7.09	15.76	Flavogallonic acid	255.4, 363.6	469.0053	0.93	C_21_H_9_O_13_	425, 301, **300**, 271	2.810 ± 0.008	6.891 ± 0.028
13	8.31	13.98	HHDP digalloyl hexose	273.0	785.0855	1.55	C_34_H_25_O_22_	483, **301**, 275, 249, 169, 125	5.603 ± 0.028	17.333 ± 0.065
14	10.21	21.04	Quercetin 3-*O*-galloyl hexoside	260.5, 359.8	615.0997	1.82	C_28_H_23_O_16_	615, 463, 301, **300**, 271, 255, 169, 151	1.337 ± 0.014	6.161 ± 0.015
15	10.37	19.3	HHDP trigalloyl hexose	279.3	937.0963	1.11	C_41_H_29_O_26_	937, 465, **301**, 275, 169, 153	2.337 ± 0.012	13.165 ± 0.018
16	10.81	21.85	Quercetin 3-*O*-galloyl hexoside	260.5, 359.8	615.0998	1.92	C_28_H_23_O_16_	615, 463, 301, **300**, 271, 255, 169, 151	tr	3.105 ± 0.008
17	10.98	23.05	Ellagic acid	252.9, 367.4	300.9991	2.24	C_14_H_5_O_8_	**301**, 257	6.591 ± 0.068	67.784 ± 0.035
**18** **	11.44	23.98	Quercetin-3-*O*-β-d-galactopyranoside (Hyperoside)	255.4, 353.6	463.0882	2.43	C_21_H_19_O_12_	463, 301, **300**, 271, 255	11.952 ± 0.029	48.01 ± 0.094
19	11.84	24.71	Kaempferol 3-*O*-galloyl hexoside	-	599.1057	2.54	C_28_H_23_O_15_	**599**, 447, 313, 285, 284, 255, 227, 169, 151	tr	tr
**20** **	11.86	24.69	Quercetin-3-*O*-β-d-glucopyranoside (Isoquercetrin)	256.7, 353.6	463.0885	3.02	C_21_H_19_O_12_	463, 301, **300**, 271, 255	10.573 ± 0.008	42.169 ± 0.074
21	12.47	26.44	Quercetin 3-*O*-pentoside	254.2, 353.7	433.0779	0.71	C_20_H_17_O_11_	433, 301, **300**, 271, 255	1.585 ± 0.011	6.558 ± 0.027
22	12.55	25.34	Quercetin galloyl hexoside	254.8, 357.0	615.0982	−0.65	C_28_H_23_O_16_	615, **301**, 179, 169, 151	0.966 ± 0.001	4.876 ± 0.011
**23** **	13.02	27.15	Kaempferol-3-*O*-β-d-galactopyranoside	265.5, 346.0	447.0935	0.49	C_21_H_19_O_11_	447, 285, **284**, 255, 227	7.124 ± 0.141	29.525 ± 0.040
24	13.29	27.09	Kaempferol 3-*O*-galloyl hexoside	-	599.1059	2.76	C_28_H_23_O_15_	**599**, 447, 313, 285, 284, 255, 227, 169, 151	tr	tr
25	13.38	26.86	Kaempferol-3-*O*-rutinoside	266.7, 350.0	593.1520	1.32	C_27_H_29_O_15_	593, **285**, 284, 227	2.917 ± 0.023	6.036 ± 0.060
**26** **	13.41	27.57	Quercetin 3-*O*-α-l-arabinofuranoside (Avicularin)	256.7, 351.0	433.0777	0.07	C_20_H_17_O_11_	433, 301, **300**, 271	4.150 ± 0.011	16.843 ± 0.055
27 *	13.58	22.92	Quercetin-3-*O*-β-rutinoside (Rutin)	254.2, 365.0	609.1461	0.92	C_27_H_29_O_16_	609, **301**, 300, 271, 255, 151	1.441 ± 0.004	tr
**28** **	13.93	27.96	Kaempferol-3-*O*-β-d-glucopyranoside	266.8, 344.8	447.0927	−0.86	C_21_H_19_O_11_	447, 285, **284**, 255, 227	24.746 ± 0.002	109.772 ± 0.31
**29** **	13.98	28.18	Quercetin-3-*O*-α-rhamnopyranoside	256.7, 348.5	447.0935	0.49	C_21_H_19_O_12_	447, 301, **300**, 271, 255	4.346 ± 0.011	19.047 ± 0.042
30	14.05	27.89	Flavogallonic acid methyl ester	261.7, 349.8	483.0207	0.30	C_22_H_11_O_13_	451, **301**, 271	tr	tr
31	14.57	28.30	Kaempferol galloyl hexoside	266.8, 347.3	599.1055	2.15	C_28_H_23_O_15_	313, **285**, 169, 151	1.256 ± 0.012	6.347 ± 0.064
**32** **	14.77	29.15	Kaempferol-3-*O*-β-xylopyranoside	264.2, 346.0	417.0828	0.25	C_20_H_17_O_10_	417, 285, **284**, 255, 227	2.703 ± 0.134	12.087 ± 0.287
**33** **	15.54	29.76	Kaempferol-3-*O*-α-arabinofuranoside (Juglanin)	264.2, 347.3	417.0829	0.40	C_20_H_17_O_10_	417, 285, **284**, 255, 227	5.420 ± 0.005	23.784 ± 0.060
**34** **	15.67	29.25	Kaempferol-3-*O*-β-glucopyranosyl (1 → 4)-α-l-rhamnopyranoside (Multiflorin B)	264.2, 344.7	593.1515	0.5	C_27_H_29_O_15_	593, **285**, 284, 227	4.629 ± 0.118	13.77 ± 0.243
**35** **	16.27	30.34	Kaempferol-3-*O*-α-l-rhamnopyranoside	256.7, 356.1	431.0983	−0.1	C_21_H_19_O_10_	431, **285**, 284, 255, 227	6.647 ± 0.004	29.891 ± 0.060
**36** **	17.41	30.50	Quercetin-3-*O*-[6-*O*-acetyl-β-d-glucopyranosyl] (1 → 4)-α-l-rhamnopyranoside	264.2, 351.0	651.1580	2.07	C_29_H_31_O_17_	651, 609, **301**, 271, 255	tr	tr
37	18.29	31.40	Quercetin p-coumaroyl hexoside	264.2, 313.3, 365.0 sh	609.1268	2.94	C_30_H_25_O_14_	609, 463, 301, **300**, 271, 255, 151	1.365 ± 0.002	6.092 ± 0.006
38 *	18.98	34.16	Quercetin	256.7, 373.7	301.0353	−0.12	C_15_H_9_O_7_	**301**, 273, 179, 151	3.859 ± 0.003	16.758 ± 0.103
**39** **	19.52	32.11	Kaempferol-3-*O*-[6‴-*O*-acetyl-β-d-glucopyranosyl] (1 → 4)-α-l-rhamnopyranoside (Multiflorin A)	263.0, 343.5	635.1626	1.32	C_29_H_31_O_16_	635, 593, 477, **285**, 257	1.818 ± 0.002	9.136 ± 0.020
**40** **	20.41	33.24	*trans*-Tiliroside	265.5, 312.1, 360.0 sh	593.1306	0.93	C_30_H_25_O_13_	593, 447, **285**, 284, 255, 227	4.905 ± 0.001	24.024 ± 0.076
41	21.32	33.98	*cis*-Tiliroside	260.5, 312.1, 360.0 sh	593.1301	0.11	C_30_H_25_O_13_	593, 447, **285**, 284, 255, 227	0.750 ± 0.002	3.073 ± 0.020
42 *	22.95	38.20	Kaempferol	265.5, 364.9	285.0404	−0.3	C_15_H_9_O_6_	**285**	4.751 ± 0.008	20.206 ± 0.051

^a^ Retention time and UV spectra from UHPLC-HRMS/MS. ^b^ Retention time, *m*/*z*, Δ, formula, and MS/MS fragmentation from UHPLC-HRMS/MS; MS/MS fragments in bold—100% intensity. ^c^ Content of compounds **3**–**13, 15,** and **17** was determined as mg gallic acid equivalents/g extract (mgGAE/gE); content of flavonoid glycosides—as mg hyperoside equivalents/g extract (mg HypE/gE). Results are presented as the mean ± standard deviation (SD). DRE—dry rose extract; EAE—EtOAc obtained after re-extraction of dry rose extract; * compounds identified with authentic standards; ** compounds confirmed by NMR; nd—not detected, nq—not quantified, tr—traces.

**Table 2:** Antioxidant potential of DRE and EAE.

Extract	DPPH (IC_50_ mg/mL)	ABTS (mM Trolox/g E)	FRAP (mM Fe^2+^/g E)
DRE	0.27 ± 0.01	1.98 ± 0.01	5.40 ± 0.14
EAE	0.16 ± 0.01	3.49 ± 0.01	13.84 ± 0.16
BHT	0.47 ± 0.03	-	8.92 + 0.08
Caffeic acid	0.068 ± 0.001	-	14.36 ± 0.01

Results are presented as mean ± standard deviation (SD). (-)—not determined.

**Table 3:** Antimicrobial activity of DRE and EAE against test pathogens.

Test Strain	Inhibition Zone (mm)				
	(+) Control		5% DMSO	DRE	EAE
*Bacillus cereus* ATCC 11778	Gentamicin 10 µg/disk	19.17 ± 0.45	NZ	11.92 ± 0.45 ^b^	10.01 ± 0.08 ^b^
*Staphylococcus aureus* ATCC 25923	Gentamicin 10 µg/disk	17.34 ± 0.48	NZ	17.08 ± 0.24 ^a^	21.86 ± 0.19 ^b^
*Staphylococcus epidermidis* ATCC 12228	Gentamicin 10 µg/disk	24.51 ± 0.42	NZ	23.67 ± 0.30 ^a^	27.99 ± 0.04 ^b^
*Propionibacterium acnes* (an isolate)	Clindamycin 2 µg/disk;	17.65 ± 0.50	NZ	21.92 ± 0.02 ^b^	25.04 ± 0.26 ^b^
*Escherichia coli* ATCC 25922	Gentamicin 10 µg/disk	18.73 ± 0.59	NZ	8.40 ± 0.29 ^b^	11.03 ± 0.27 ^b^
*Pseudomonas aeruginosa* ATCC 27853	Gentamicin 10 µg/disk	17.38 ± 0.77	NZ	9.08 ± 0.21 ^b^	10.87 ± 0.51 ^b^
*Candida albicans* ATCC10231	Nystatin 100 units/disk	22.0 ± 0.09	NZ	NZ	NZ

Note: NZ—no inhibition zone. Values are expressed as mean ± standard deviation (SD). Statistical analysis was performed using ANOVA and post hoc Tukey test: ^a^—nonsignificant (*p* > 0.05); ^b^—significant (*p* < 0.01).

**Table 4:** MICs of DRE and EAE determined by the agar microdilution method.

Test Strain	MIC, mg/mL	
	DRE	EAE
*Bacillus cereus* ATCC 11778	5	2.5
*Staphylococcus aureus* ATCC 25923	10	5
*Staphylococcus epidermidis* ATCC 12228	20	5
*Propionibacterium acnes* (an isolate)	10	5
*Escherichia coli* ATCC 25922	2.5	10
*Pseudomonas aeruginosa* ATCC 27853	20	5

## Data Availability

Data are contained within the article and Supplementary Materials.

## Supplementary Materials

The following supporting information can be downloaded at: https://www.mdpi.com/article/10.3390/molecules28227666/s1, Figure S1. Structures of isolated flavonoids; Table S1: ^1^H NMR data of quercetin glycosides; Table S2: ^1^H NMR data of kaempferol glycosides; Table S3: Total phenolic (TPC) and total flavonoid (TFC) contents and antioxidant activity (DPPH, ABTS, and FRAP) of dry rose extract (DRE) and ethyl acetate fraction (EAE).
