# Supplementary material for: Phytochemical Profile, Antioxidant Potential, Antimicrobial Activity, and Cytotoxicity of Dry Extract from Rosa damascena Mill
Source: Molecules. 2023 Nov 19;28(22):7666. doi: 10.3390/molecules28227666 (PMC10674922; doi:10.3390/molecules28227666)
Supplement: Supplementary file 1 [file molecules-28-07666-s001.zip › molecules-2639499-supplementary.pdf]

SUPPLEMENTARY MATERIAL

Phytochemical Profile, Antioxidant Potential, Antimicrobial Activity and Cytotoxicity of Dry Extract from *Rosa damascena* Mill

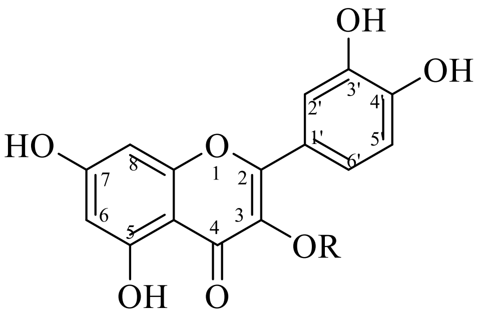

18 R = Gal
20 R = Glc
26 R = Ara
29 R = Rha
36 R = Glc (1→4) Rha, R' = Ac

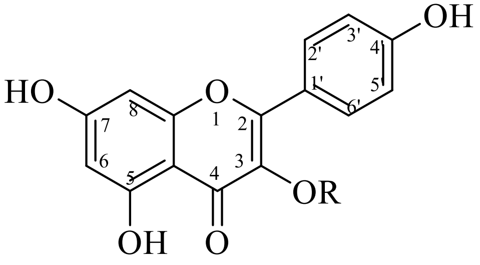

23 R = Gal
28 R = Glc
32 R = Xyl
33 R = Ara
34 R = Glc (1→4) Rha, R' = H
35 R = Rha
39 R = Glc (1→4) Rha, R' = Ac
40 R = 6″-(E)-p-Coumaroyl-Glc

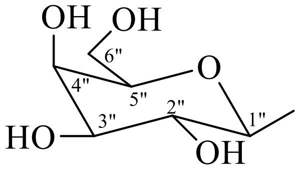

Gal

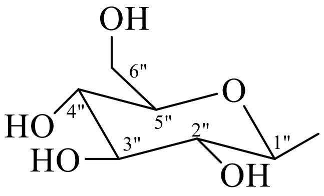

Glc

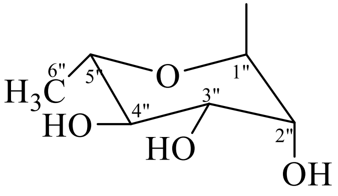

Rha

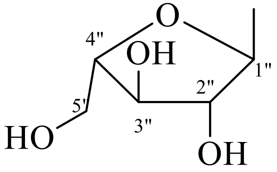

Ara

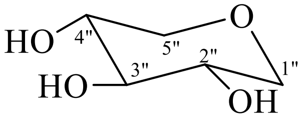

Xyl

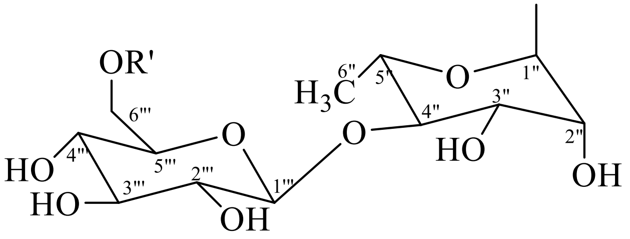

Glc (1→4) Rha

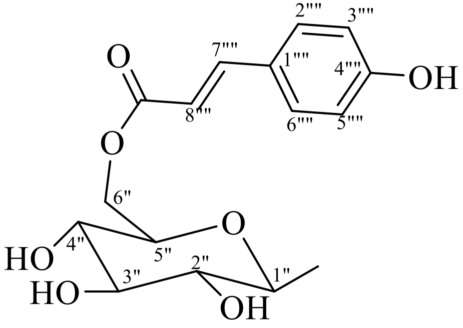

6″-(E)-p-Coumaroyl-Glc

Figure S1. Structures of isolated flavonoids

Table S1. ^{1}H NMR data of quercetin glycosides (600 MHz, CD_3OD, δ in ppm, multiplicity, *J* constants)

	**18**	**20**	**26**	**29**	**36**
H-6	6.20 d (2.1)	6.20 d (2.1)	6.19 d (1.9)	6.19 d (2.0)	6.17 d (2.1)
H-8	6.40 d (2.1)	6.39 d (2.1)	6.38 d (1.9)	6.36 d (2.0)	6.34 d (2.1)
H-2’	7.84 d (2.2)	7.70 d (2.2)	7.53 d (2.1)	7.32 d (2.1)	7.32 d (2.2)
H-5’	6.86 d (8.2)	6.86 d (8.2)	6.90 d (8.3)	6.90 d (8.0)	6.91 d (8.0)
H-6’	7.58 dd (2.2, 8.2)	7.58 dd (2.2, 8.2)	7.49 dd (2.1, 8.3)	7.30 dd (2.1, 8.0)	7.27 dd (2.2, 8.0)
H-1”	5.17 d (7.7)	5.26 d (7.6)	5.47 brs	5.33 d (1.5)	5.35 d (1.5)
H-2”	3.81 dd (7.7, 9.1)	3.47 dd (7.6, 9.0)	4.33 br d (3.0)	4.20 dd (1.4, 3.4)	4.20 dd (1.5, 3.4)
H-3”	3.55 dd (3.2, 9.1)	3.42 t (9.0)	3.91 dd (3.0, 5.1)	3.74 dd (3.4, 9.4)	3.93 dd (3.4, 9.1)
H-4”	3.84 br d (3.2)	3.34 t (9.0)	3.86 m	3.33 m	3.50 t (9.1)
H-5”	3.46 br t (6.1)	3.21 m	3.49 m (2H)	3.41 m	3.36 m
H-6”	3.63 dd (6.1, 11.2)	3.70 dd (2.3, 12.1)		0.93 d (6.5)	0.95 d (6.7)
	3.54 dd (6.1, 11.2)	3.56 dd (5.6, 12.1)			
H-1”’					4.48 d (7.7)
H-2”’					3.19 dd (7.7, 9.1)
H-3”’					3.33 t (9.1)
H-4”’					3.26 m
H-5”’					3.42 m
H-6”’					4.35 dd (2.5, 12.0)
					4.18 dd (5.4, 12.0)
OAc					2.01 s

Table S2. ^{1}H NMR data of kaempferol glycosides (600 MHz, CD_3OD, δ in ppm, multiplicity, *J* constants)

	**23**	**28**	**32**	**33**	**34**	**35**	**39**	**40**
H-6	6.19 d (2.1)	6.2 d (2.0)	6.18 d (2.1)	6.19 d (2.1)	6.19 d (2.1)	6.20 d (2.0)	6.19 d (2.1)	6.13 d (2.1)
H-8	6.38 d (2.1)	6.4 d (2.0)	6.38 d (2.1)	6.38 d (2.1)	6.36 d (2.1)	6.37 d (2.0)	6.36 d (2.1)	6.32 d (2.1)
H-2'/H-6'	8.10 d (8.9)	8.06 d (9.0)	8.04 d (9.0)	7.96 d (9.1)	7.76 d (8.8)	7.77 d (8.9)	7.75 d (8.8)	7.99 d (8.8)
H-3'/H-5'	6.88 d (8.9)	6.88 d (9.0)	6.88 d (9.0)	6.92 d (9.1)	6.92 d (8.8)	6.94 d (8.9)	6.93 d (8.8)	6.82 d (8.8)
H-1"	5.12 d (7.7)	5.25 d (7.5)	5.17 d (6.9)	5.47 brs	5.37 d (1.5)	5.37 d (1.8)	5.35 d (1.5)	5.24 d (7.4)
H-2"	3.79 dd (7.7, 9.1)	3.43 dd (7.5, 9.1)	3.48 dd (6.9, 8.2)	4.32 brd (3.1)	4.24 dd (1.5, 3.4)	4.22 dd (1.8, 3.4)	4.20 dd (1.5, 3.4)	3.45 dd (7.5, 9.1)
H-3"	3.53 dd (3.2, 9,1)	3.41 t (9.1)	3.39 t (8.2)	3.91 dd (3.1, 5.2)	3.94 dd (3.4, 9.1)	3.72 dd (3.4, 9.4)	3.93 dd (3.4, 9.1)	3.35 m
H-4"	3.82 br d (3.2)	3.32 t (9.1)	3.48 m	3.79 m	3.54 t (9.1)	3.32 m	3.50 t (9.1)	3.44 m
H-5"	3.43 br t (5.5)	3.20 m	3.76 dd (4.7, 11.2)	3.47 m (2H)	3.38 m	3.32 m	3.36 m	3.44m
			3.09 dd (9.0, 11.2)					
H-6"	3.63 dd (5.5, 11.3)	3.69 dd (2.5, 12.1)			1.01 d (6.7)	0.93 d (6.5)	0.95 d (6.7)	4.29 dd (2.5, 11.8)
	3.54 dd (5.5, 11.3)	3.52 dd (5.6, 12.1)						4.18 dd (6.7, 11.8)
H-1'''					4.49 d (7.7)		4.47 d (7.7)	
H-2'''					3.19 dd (7.7, 9.1)		3.19 dd (7.7, 9.1)	
H-3'''					3.35 t (9.1)		3.33 t (9.1)	
H-4'''					3.37 m		3.26 m	
H-5'''					3.22 m		3.42 m	
H-6'''					3.80 dd (2.5, 12.0)		4.36 dd (2.5, 12.0)	
					3.66 dd (5.4, 12.0)		4.18 dd (5.4, 12.0)	
OAc							2.01 s	
H-2''''/H-6''''								7.32 d (8.8)
H-3''''/H-5''''								6.79 d (8.8)
H-7''''								7.39 d (15.9)
H-8''''								6.07 d (15.9)

Table S3. Total phenolic (TPC) and total flavonoid (TFC) contents and antioxidant activity (DPPH, ABTS and FRAP) of dry rose extract (DRE) and ethyl acetate fraction (EAE)

Extract	TPC [mg GAE/g E]	TFC [mg RE/g E]	DPPH [IC_{50} mg/mL)	ABTS [mM Trolox/g E]	FRAP [mM Fe^{2+}/g E]
**DRE**	212.19±3.43	135.28±1.77	0.27±0.01	1.98±0.01	5.40±0.14
**EAE**	680.48±2.48	482.26±1.82	0.16±0.01	3.49±0.01	13.84±0.16
**BHT**	-	-	0.47±0.03	-	8.92+0.08
**Caffeic acid**	-	-	0.068±0.001	-	14.36±0.01
